# Structured postmortem genetic counselling and testing in sudden unexplained death among young adults in the Netherlands: results and implications regarding hereditary risk

**DOI:** 10.1007/s12687-026-00946-z

**Published:** 2026-10-17

**Authors:** Tess M. Wemeijer, Leonie A. Verbaan, Wilma L.J.M. Duijst, Yvonne M. Hoedemaekers, Imke Christiaans

**Affiliations:** 1 Public Health Service, Department of Forensic Medicine, GGD IJsselland, Zwolle, the Netherlands; 2 Faculty of Law, Maastricht University, Maastricht, the Netherlands; 3 Department of Genetics, Radboud University Medical Centre, Nijmegen, the Netherlands; 4 Department of Genetics, University Medical Centre Groningen, University of Groningen, Groningen, the Netherlands

**Keywords:** Sudden unexplained death, Sudden cardiac death, Postmortem genetic testing, Hereditary cardiovascular disorder, Genetic counselling

## Abstract

**Introduction:**

Sudden unexplained death (SUD) in young adults may result from hereditary cardiovascular disorders that remain undetected even after conventional autopsy. Routine postmortem genetic testing may identify these disorders, improving determination of the cause of death while providing diagnostic and preventive opportunities for surviving blood relatives.

**Methods:**

Between September 2022 and October 2025, 100 cases of SUD in young adults in the Netherlands were investigated using the NODOV (Nader Onderzoek naar de DoodsOorzaak bij (jong)Volwassenen) procedure. Genetic counselling was offered to surviving blood relatives in 50 cases where conventional autopsy findings suggested an inherited cardiovascular disorder. Conventional autopsy findings, medical history and family history guided phenotype-directed genetic testing using targeted gene panel analysis following whole-exome/genome sequencing.

**Results:**

Genetic testing was successfully performed in 46 out of 50 cases and identified pathogenic variants in six cases involving the CACNA1C, BAG3, MYBPC3, LDL-R, KCNJ2, and PKD1 genes. Pretest genetic counselling proved important in guiding genetic testing in 10% of all cases and 50% of genetically confirmed cases. Pathogenic variants were identified in both structurally abnormal and autopsy-negative cases. Families received tailored genetic counselling and recommendations for clinical follow-up based on predictive genetic testing. Among the 44 families without a genetic diagnosis, 43 were advised to undergo clinical cardiovascular screening.

**Conclusion:**

This study highlights the importance of a structured multidisciplinary postmortem investigation, such as the NODOV procedure, integrating pretest genetic counselling and tailored postmortem genetic testing. This approach may improve determination of cause of death (particularly in autopsy-negative cases), aid in the identification of hereditary cardiovascular disorders and facilitate preventive strategies for at-risk blood relatives.

**Supplementary Information:**

The online version contains supplementary material available at https://doi.org/10.1007/s12687-026-00946-z.

## Introduction

Sudden unexplained death (SUD) in young adults is a rare event that raises questions regarding the underlying cause of death, the presence of hereditary cardiovascular disorders, and opportunities for prevention (Eckart et al. 2011). SUD refers to a sudden and unexpected death in which no definitive cause is identified during the initial forensic investigation, irrespective of whether a conventional autopsy has been performed. When a cardiac cause of sudden death is suspected, SUD casus are referred to as sudden cardiac death (SCD) (Fellman et al. 2019). Causes of SCD include structural cardiac abnormalities such as cardiomyopathies, coronary artery disease (CAD) and valvular heart disease (Abbas et al. 2023; Arava et al. 2025; Katritsis et al. 2016). Sudden arrhythmic death syndrome (SADS) represents a subset of SCD describing sudden death in individuals with negative autopsy findings and negative toxicology (Fellman et al. 2019; Mellor et al. 2014; Stiles et al. 2020) or a hereditary arrhythmogenic disorder or concealed cardiomyopathy is either suspected or genetically confirmed (Monda et al. 2026).

Previous studies have reported an average incidence of SUD between 0.75 and 11.9/100,000 person-years among individuals younger than 40 years (Couper et al. 2020; Fellmann et al. 2019)and SCD in approximately 70% of cases (Hofer et al. 2014; van der Werf et al. 2010).

Conventional autopsy remains the gold standard for determining the cause of death; however, some cases remain unexplained despite conventional autopsy (Lynge et al. 2024). The cause of death remains unexplained in approximately 30–40% of SCD cases in individuals aged 29–48 years, in whom a structurally normal heart was reported during conventional autopsy (Abbas et al. 2023; Bagnall et al. 2016; Guo et al. 2021; Stiles et al. 2020).

Consequently, surviving relatives may be left without clear information regarding the cause of death or its implications, potentially resulting in uncertainty and emotional distress. In addition, surviving blood relatives may be at risk of carrying undetected hereditary disorders, potentially exposing them to health risks that could otherwise be identified and treated through timely genetic and medical assessment (Deignan et al. 2023).

Prior studies demonstrated that postmortem genetic testing identified pathogenic or likely pathogenic variants associated with the cause of death in approximately 22.1% of SUD cases (range, 20–23%) (Ponmani et al. 2026). Among cases classified as SADS, the overall pooled diagnostic yield of postmortem genetic testing was reported to be 11% when both channelopathy- and cardiomyopathy-associated genes were analysed (Monda et al. 2026). Previous research showed that hereditary cardiomyopathies and primary arrhythmic causes were more prevalent among younger individuals under the age of 35, whereas SCD in older individuals over 35 years of age was predominantly attributed to CAD and myocardial infarction (Fellmann et al. 2019; Landry et al. 2017; Li Tan et al. 2025; Stiles et al. 2020).

This age-dependent shift in etiology may suggest a greater contribution of environmental factors and gene–environment interactions in older individuals, particularly in the pathogenesis of CAD. Nevertheless, postmortem genetic testing remains clinically valuable across age groups, as the identification of pathogenic or likely pathogenic variants can facilitate the detection of at-risk blood relatives and enable targeted cascade screening, preventive interventions, and long-term clinical surveillance, thereby potentially reducing the incidence of subsequent sudden deaths within affected families (Lynge et al. 2024).

In 2022, a new postmortem procedure for young adults aged 18–45 years was implemented in the Netherlands, partly based on European recommendations (Basso et al. 2017; Fellmann et al. 2019). This multidisciplinary procedure, known as NODOV (Nader Onderzoek naar de DoodsOorzaak bij (jong)Volwassenen; in English translated to further investigation into the cause of death amongst adults), integrates multiple postmortem diagnostic modalities, including toxicological analysis, computed tomography (CT) imaging, conventional autopsy, postmortem genetic testing and genetic counselling (Wemeijer et al. 2025).

This study aimed to evaluate the outcomes of the Dutch national NODOV protocol incorporating postmortem genetic testing and genetic counselling after sudden unexplained death in young adults (< 45 years), and to assess its clinical implications for surviving blood relatives.

## Methods

### Study design

This multicentre prospective cohort study was conducted in the Netherlands between September 2022 and October 2025. During this study period, 100 individuals aged 18–45 years who experienced sudden and unexplained death presumed to be of natural origin were included in the NODOV study (hereafter also referred to as the NODOV procedure).

Eligible cases were identified by the attending forensic physician. Forensic physicians in participating regions in the Netherlands had been informed about the study procedures and inclusion criteria. The deceased’s spouse or relative was approached at the scene or, when necessary, located by the police to obtain informed consent for participation. Consent was required before inclusion in the NODOV procedure and was obtained or declined within 24 h of the forensic investigation. Postmortem CT and conventional autopsy were performed within 24 h, with results typically available within 24 h when brain autopsy was not performed. Blood samples for toxicological analysis were collected and submitted within the same timeframe, with results typically available within two weeks.

Each case was evaluated by a multidisciplinary team comprising of the attending forensic physician, the NODOV case manager (a forensic physician), a radiologist specialising in post-mortem CT, a toxicologist, a pathologist with thorough knowledge of cardiac pathology (de Noronha et al. 2014; Stiles et al. 2020) and a clinical geneticist. The composition of the team, except for the NODOV case manager, varied according to case-specific requirements. Additional specialist expertise was sought when indicated. Following review of the circumstances of death, post-mortem CT findings, toxicology results and conventional autopsy findings, the multidisciplinary team reached a consensus regarding the cause of death and the likelihood of an underlying hereditary disorder.

The indication for post-mortem genetic testing was determined after integration of the findings from the other post-mortem investigations performed within the NODOV procedure. Surviving blood relatives were referred by their general practitioner for genetic counselling when the findings suggested a possible hereditary condition (e.g. evidence of cardiomyopathy or premature CAD), when the cause of death remained unexplained, or when suspicion of a genetic predisposition persisted despite completion of the post-mortem investigation.

In the Netherlands, health insurance coverage ceases upon death. Within the NODOV study, all costs associated with the post-mortem investigation were covered, except for genetic counselling and genetic testing. The costs of genetic counselling and genetic testing of the deceased were reimbursed through the health insurance of an eligible living relative (Stiles et al. 2020), although in some cases a personal financial contribution was required because of the deductible excess. Genetic counselling of the blood relative was scheduled 4–6 weeks after definitive autopsy results and subsequent genetic testing generally took a few months. (Genetic) test results were communicated to relatives by a genetic counsellor during an in-person or video consultation and were confirmed in writing, including advice for genetic or cardiovascular testing for the counselled relative and other eligible blood relatives.

### Genetic counselling and genetic testing

Genetic testing was preceded by pretest genetic counselling of a surviving blood relative of the deceased. During genetic counselling, the genetic counsellor discussed information regarding the deceased’s medical history, family history, potential heritable conditions, and the possible outcomes and limitations of genetic testing. Additional consent was requested to perform genetic testing. Postmortem genetic testing was performed on freshly frozen, or paraffin embedded tissue of the spleen obtained from the deceased during autopsy. Genetic testing was performed in one of the university-affiliated DNA laboratories. Guided by the pathological findings from the conventional autopsy, suspected underlying disease mechanisms, and patient and family history, targeted gene panels were selected to optimize diagnostic yield (Stiles et al. 2020). Several gene panels were available: an arrhythmia gene panel, a cardiomyopathy gene panel, and a dyslipidaemia gene panel. Additional phenotype-directed panels were applied when other heritable disease categories were suspected. Also, in a select number of cases family history showed a known genetic cause. In these cases, genetic testing focused on the familial variant. Molecular analyses were predominantly performed using whole-exome sequencing (WES) with subsequent filtering on respective gene panels (Stiles et al. 2020). As the study was conducted over multiple years and across different laboratories, different versions of specific gene panels were used, meaning gene content could vary mildly. Genetic variants were classified into pathogenicity classes according to the American College of Medical Genetics and Genomics (ACMG) guidelines (Richards et al. 2015). Clinically relevant findings, including pathogenic and likely pathogenic variants, as well as variants of uncertain significance (VUS) with strong molecular or clinical suspicion, were documented for potential familial evaluation. Stiles et al. (2020) recommend that “care should be taken in conveying test results of uncertain significance” (Bates et al. 2019; Burns et al. 2017), which is why only VUS with strong molecular or clinical suspicion were shared with surviving relatives during genetic counselling. In selected cases genetic testing was performed in an affected relative since postmortem genetic testing is known to have a higher frequency of false negative results and used techniques on tissue do not allow for copy number variant analysis.

### Outcomes

The primary outcome measures were (a) the identification of pathogenic or likely pathogenic genetic variants associated with the cause of death, and (b) cardiovascular screening advice for blood relatives of gene-elusive deceased patients.

### Data analysis

Descriptive analyses were performed. Data are presented as percentages, ranges, and qualitative summaries. No inferential statistical analyses were conducted.

## Results

### Study population

**Fig. 1:**
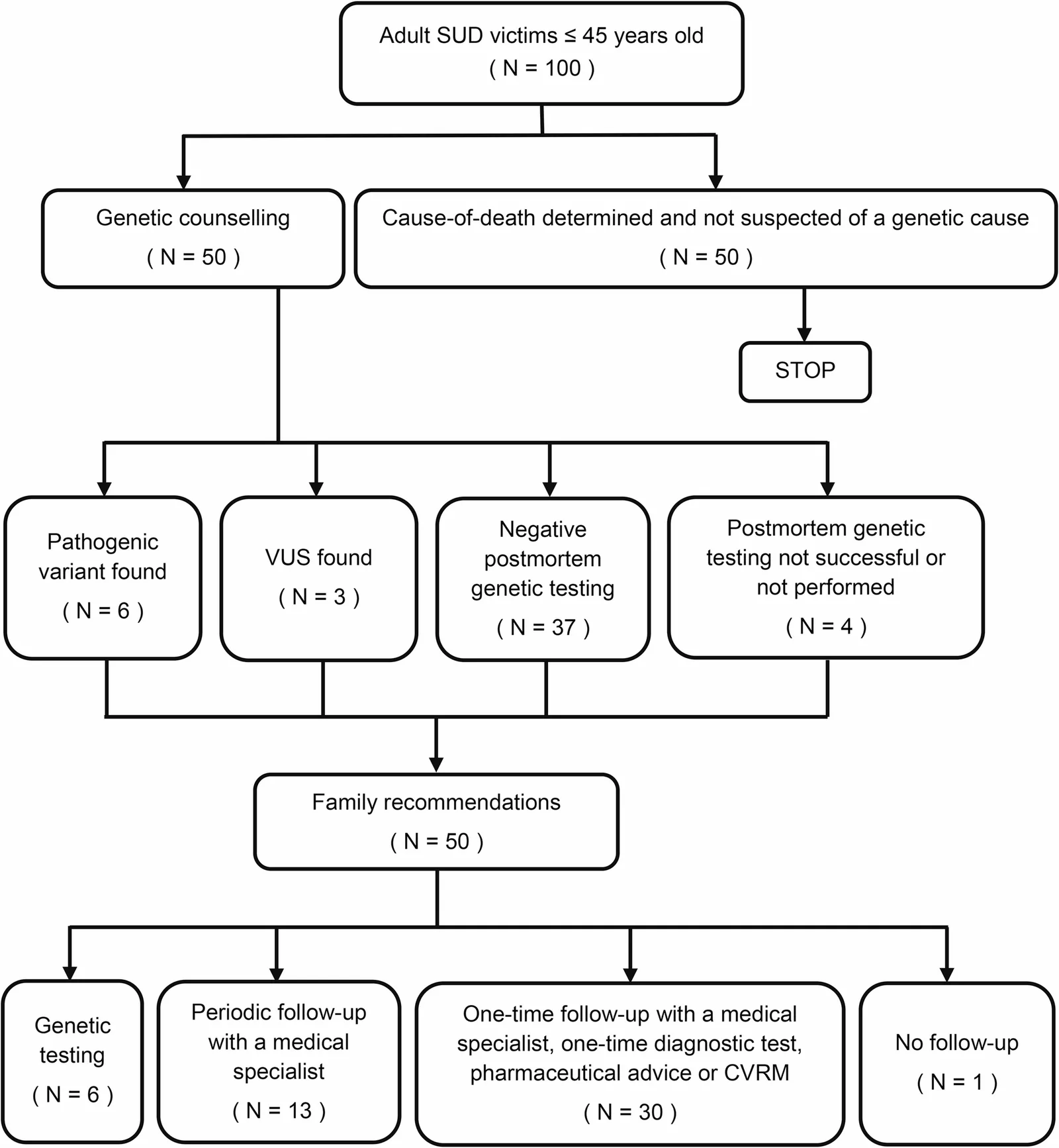
Flowchart of 50 selected cases that were eligible for genetic counselling and postmortem genetic testing: Test results and family recommendations given. The highest level of advice given to the 50 families by a genetic counsellor is recorded under family recommendations, irrespective of whether families decided to follow through and if multiple recommendations were given within one family

In 50 out of 100 SUD cases (50%) included in the NODOV study, either no cause of death was established through the other postmortem examination modalities (excluding postmortem genetic testing) within the NODOV procedure, or the cause of death was suggestive for an underlying hereditary disorder. Consequently, surviving blood relatives in these 50 cases were offered pre-test genetic counselling. See Fig. 1 for further details. The study population was comprised of 10 females (20%) and 40 males (80%), with an age range of 19–45 years.

### Conventional autopsy findings

Conventional autopsy was performed in 48 out of 50 cases. In two out of 50 cases postmortem CT imaging showed a clear cause of death, making conventional autopsy redundant. In 13 out of 48 cases no cause of death could be established during conventional autopsy, and these were classified as autopsy-negative. There were (relevant) findings in 35 out of 48 conventional autopsy cases: CAD was the most common observation in sudden young deaths, with evidence of myocardial infarction in 14 cases. Myocardial infarction due to CAD was most common in the older age groups (37–45 years). CAD without certain evidence of myocardial infarction (negative lactate dehydrogenase staining) was described in seven cases. Cardiomyopathy was described in eleven cases. In two of these cases both CAD and cardiomyopathy were described. Other vascular disorders (type-A-dissection and brain aneurysms) were described in three cases and pulmonary embolism in one case. Myocardial infarction with nonobstructive coronary arteries (MINOCA), and multiorgan failure (MOF) were each described in one case. See Supplementary Appendix 1 for further details.

### Postmortem genetic testing results

Of the 50 families receiving genetic counselling, genetic testing was successfully performed in 46 families. Among the 46 successfully tested cases, a pathogenic variant was identified in six cases (approximately 13%). Pathogenic variants were identified in the *CACNA1C*, *BAG3*, *MYBPC3*, *LDL-R*, *KCNJ2* and *PKD1* genes. The conventional autopsy findings were consistent with the detected genetic cause: The two cases with a pathogenic variant in a gene related to channelopathies (*CACNA1C*, *KCNJ2*) had negative conventional autopsies. The two cases with a pathogenic variant in a cardiomyopathy gene (*BAG3*, *MYBPC3*) had visible signs of cardiomyopathy at autopsy. The one case with a pathogenic variant in a gene related to familial hypercholesterolemia (*LDL-R*) had significant CAD. The one case with a pathogenic gene related to autosomal dominant polycystic kidney disease (*PKD1*) had polycystic kidneys, a polycystic liver, and a brain aneurysm. In three additional cases a VUS was found with a strong molecular or clinical suspicion. See Supplementary Appendix 1 for further details.

### Genetic counselling and family recommendations

One or more surviving blood relatives participated in genetic counselling in all 50 eligible cases, allowing discussion of genetic testing and family recommendations. Pretest genetic counselling resulted in tailored genetic testing or tailored family counselling in five cases (10%) and three out of six positive genetic cases (50%). In one family, the genetic cause had been identified in their extended family before the sudden and unexplained death happened, leading to targeted variant testing in the deceased. In another family, although initial postmortem genetic testing of the deceased was negative, genetic counselling identified blood relatives with cardiac abnormalities, resulting in genetic testing of an affected relative. As a result, the blood relatives detected pathogenic variant could later be confirmed in a different tissue specimen of the deceased. In one case genetic testing was not performed since the genetic variant had already been known before death. Family recommendations and genetic testing of blood relatives was performed. In two families with CAD, genetic testing was performed in one or more affected surviving blood relatives. Results were negative. Testing of blood-derived DNA from the relatives enabled copy number variant analysis of the gene panel, which could not be performed using DNA extracted from post-mortem tissue. Genetic test results enabled personalized follow-up recommendations for surviving blood relatives, ensuring that periodic clinical surveillance was reserved for those with an increased risk of hereditary disease, while others were advised a one-time evaluation.

The six families of the SUD victims in which a pathogenic variant was found were advised to undergo genetic testing and/or further examination. Cascade genetic testing was recommended to identify surviving blood relatives at risk for heritable disease. In one case in which a pathogenic variant was found, the family decided not to go through with genetic testing. One reason being that the deceased individual had no children.

Three VUS were found with strong molecular or clinical suspicion involving the *LDL-R* and *COL3A1* genes.

Forty-three out of 44 families of deceased individuals with a negative genetic test result (including VUS) were mostly given recommendations based on clinical guidelines. Families received a family letter with instructions at which age testing should start (and finish) and what type of testing was recommended. In cases with a stronger suspicion of a genetic cause, e.g. when a cardiomyopathy was detected at autopsy, periodic follow-up with a medical specialist was recommended. In gene-elusive CAD cases cardiovascular risk management (CVRM) was advised. In gene-elusive autopsy-negative cases mostly a one-time extensive cardiac and/or vascular evaluation was recommended. In one case pharmaceutical advice was given to female family members. In another case family members were advised to undergo a medication provocation test. Finally, in one family no recommendations were given as the deceased was gene-elusive and a potential toxicological cause of death was also present, making an inheritable cause of death unlikely. See Fig. 1 for further details. The highest level of advice given to the families by the genetic counsellor is recorded in the flowchart, irrespective of whether families decided to follow through, or multiple recommendations were given within one family.

## Discussion

Genetic counselling was attended by one or more surviving blood relatives in all 50 cases in which an inherited cause of death was suspected. Forty-six out of 50 SUD cases (92%) successfully underwent postmortem genetic testing. Six out of these 46 cases (approximately 13%) had a positive genetic test result; four out of 33 autopsy-positive cases (approximately 12.1%) and two out of thirteen autopsy-negative cases (approximately 15.4%).

Previous research demonstrated that postmortem genetic testing could identify pathogenic or likely pathogenic variants associated with the cause of death in approximately 22.1% of SUD cases (range, 20–23%) (Ponmani et al. 2026). No distinction was made between the different categories, such as SCD and SADS, or positive and negative autopsies. It is therefore not possible to explain with certainty why overall diagnostic yield was lower in this study versus previous research (respectively, approximately 13% versus 22.1%). This study solely offered genetic counselling and postmortem genetic testing (including only relevant cardiovascular-related gene panels) in cases suspicious of a heritable cardiovascular disorder, possibly resulting in a lower number of included cases and more precision genetic testing. Furthermore, SUD cases were not excluded from this study after comprehensive postmortem investigation was performed, despite an underlying toxicological cause; further resulting in a lower diagnostic yield.

However, previous research reported that among SADS cases (i.e. autopsy-negative cases or autopsy findings with unknown significance), the overall pooled diagnostic yield of postmortem genetic testing was reported to be between 6 and 11%, depending on whether channelopathy- and cardiomyopathy-associated genes were analysed separately or combined (Monda et al. 2026). The diagnostic yield in autopsy-negative cases was higher in this study (approximately 15.4%). This may be due to the small sample size. Collectively, these findings support the additional diagnostic value of genetic counselling and postmortem genetic testing in the evaluation of young sudden unexplained death.

### Strengths

A major strength of this study is the novel standardised offer of comprehensive postmortem investigations, provided free of charge to families since 2022 through the NODOV procedure (Wemeijer et al. 2025). Transportation, conventional autopsy, toxicology analyses, and postmortem CT imaging were provided without financial burden to surviving blood relatives, while genetic testing and counselling were largely reimbursed through health insurance (except the deductible excess). By reducing socioeconomic barriers to participation, this approach likely enhanced the representativeness of the study cohort. In addition, participation was entirely voluntary, and surviving blood relatives retained full autonomy regarding consent for conventional autopsy and postmortem genetic testing, strengthening the ethical integrity of the study. The study focused on young adults in whom the likelihood of identifying hereditary causes of death is higher, as acquired cardiovascular disease due to lifestyle and/or environmental factors becomes increasingly more prevalent after age 45 (Li Tan et al. 2025).

The genetic testing strategy was tailored rather than protocolized and preceded by pretest genetic counselling. Gene selection was guided by conventional autopsy findings, family history, and known familial variants. In selected families, testing of affected surviving blood relatives enabled better testing properties and decreased false negative findings in tissue samples of the deceased, thereby increasing diagnostic yield. An additional strength is the integration of genetic counselling into the postmortem investigation. In five cases (10%) and three out of six positive genetic cases (50%) pretest genetic counselling was key to provide adequate care to the families. Besides the mentioned effects on testing strategy, pre-test genetic counselling is essential when hereditary disease is suspected, as it informs surviving blood relatives about inheritance patterns, clinical implications, psychosocial consequences, and options for cascade genetic testing in case of positive results. Identification of a pathogenic variant allows early detection and timely management of asymptomatic carriers at risk of arrhythmias, cardiomyopathies, premature CAD, heart failure, myocardial infarction, or sudden death, thereby reducing morbidity and mortality. Surviving blood relatives were offered tailored follow-up recommendations based on conventional autopsy, genetic testing and family history. Depending on the estimated hereditary risk, family recommendations ranged from one-time diagnostic testing, periodic cardiovascular risk management (CVRM) by a general practitioner, one-time extensive follow-up with a medical specialist (most often cardiological evaluation, including electrocardiography, echo-cardiography and exercise testing) to repeated periodic follow-up with a medical specialist. Such tailored follow-up strategies account for age-dependent disease expression and may reduce delayed or missed diagnoses. Families were respected in their decision to decline or defer recommended follow-up actions. The highest level of advice provided was recorded irrespective of uptake. See Fig. 1 and Supplementary Appendix 1.

This study further highlights limitations of conventional autopsy alone. Channelopathies and other hereditary arrhythmia syndromes may leave no structural abnormalities detectable at conventional autopsy, emphasizing the complementary role of molecular investigations in sudden unexplained death.

### Limitations

The relatively small sample size limits generalizability. Diagnostic yield was possibly influenced by the composition of available gene panels and current limitations in variant interpretation. Variants of unknown significance may later prove clinically relevant as genomic knowledge evolves. The presence of a VUS with strong molecular or clinical suspicion was shared with surviving blood relatives for they may prove to be a pathogenic variant in the future.

Importantly, genetic testing within the NODOV study was contingent on consent for conventional autopsy. When surviving blood relatives declined conventional autopsy, genetic testing was not performed, potentially resulting in missed hereditary diagnoses. This requirement may also have reduced inclusiveness, as some surviving blood relatives may consider conventional autopsy too invasive while still accepting minimally invasive genetic testing approaches. Precise data regarding this matter falls outside the scope of this study. Future protocols should therefore evaluate whether postmortem genetic testing can be offered independently of full conventional autopsy.

Current gene panels also remain incomplete. Novel disease-associated variants continue to be identified, and future sequencing technologies may improve detection of currently unrecognized pathogenic mechanisms. Previous studies have shown that re-evaluation over time can yield additional diagnoses, with most new findings emerging within five years after initial investigation (Semsarian and Medi 2025). The recommendation for families with persistent suspicion of hereditary disease to seek re-evaluation after several years is therefore justified and consistent with existing evidence. The findings additionally reflect the increasing recognition that hereditary cardiomyopathies, channelopathies, and familial hypercholesterolemia are not always purely monogenic disorders with straightforward Mendelian inheritance patterns. Contemporary genetic research suggests that disease susceptibility may result from complex interactions between multiple genetic variants and environmental or acquired factors, including obesity, diabetes, medication exposure, and physiological stress (Li Tan et al. 2025). Further research is therefore warranted to understand how genetic predisposition interacts with external modifiers in SUD.

Overall, these findings highlight the clinical and forensic value of the NODOV procedure as a structured multidisciplinary framework for investigating SUD in young adults in the Netherlands. Beyond improved determination of cause of death, the structured NODOV procedure creates opportunities for prevention among surviving blood relatives through targeted cascade genetic testing and individualised clinical follow-up. Future research should focus on larger prospective cohorts with standardized postmortem and genetic protocols, long-term follow-up of screened surviving blood relatives, and the evaluation of minimally invasive and cost-effective postmortem genetic testing strategies. At the policy level, broader implementation of standardized postmortem procedures incorporating genetic counselling and targeted genetic testing, such as the NODOV procedure, may improve identification and prevention of hereditary causes of SCD.

## Conclusion

This study demonstrates the added value of the structured NODOV procedure, which integrates conventional autopsy, postmortem CT imaging, toxicological analysis, genetic counselling, and tailored postmortem genetic testing in the investigation of young sudden unexplained death in the Netherlands. Pathogenic variants were identified in approximately 13% of successfully tested cases, including individuals with negative conventional autopsy findings, highlighting the complementary role of postmortem genetic testing in improving cause-of-death determination and identifying hereditary cardiovascular disorders. By combining comprehensive postmortem evaluation with family- and phenotype-directed genetic testing, the structured NODOV procedure extends the scope of postmortem investigation beyond establishing the cause of death, enabling targeted (pretest) genetic counselling, cascade screening, and individualized follow-up of at-risk blood relatives. Pretest genetic counselling of blood relatives proved essential to tailor genetic testing and provide adequate genetic care to the families.

These findings support the inclusion of genetic counselling and postmortem genetic testing as an integral component of structured sudden unexplained death investigations in young adults, with the potential to improve forensic diagnosis and facilitate preventive management aimed at reducing adverse cardiovascular events in affected families.

## Supplementary Information

Below is the link to the electronic supplementary material.

Supplementary Material 1 (DOCX 44.2 KB)

## Data Availability

All data supporting the findings of this study are available within the paper or its Supplementary Information. Addition information is found in previous publicated articles of this PhD-traject of available upon request.

## References

[CR1] Abbas R, Abbas A, Khan TK, Sharjeel S, Amanullah K, Irshad Y (2023) Sudden cardiac death in young individuals: a current review of evaluation, screening and prevention. J Clin Med Res 15(1):1–9. 10.14740/jocmr4823 PMC9881489 36755763

[CR2] Arava S, Goswami A, Pandit JN et al (2025) Sudden cardiac death in young: A cardiac-focused autopsy and molecular study to identify the cause. Indian Heart J 77(5):367–374. 10.1016/j.ihj.2025.06.007 PMC12675819 40617375

[CR3] Bagnall RD, Weintraub RG, Ingles J, Duflou J, Yeates L, Lam L et al (2016) A prospective study of sudden cardiac death among children and young adults. N Engl J Med 374:2441–2452. 10.1056/NEJMoa1510687 27332903

[CR4] Basso C, Aguilera B, Banner J et al (2017) Guidelines for autopsy investigation of sudden cardiac death: 2017 update from the Association for European Cardiovascular Pathology. Virchows Arch 471:691–705. 10.1007/s00428-017-2221-0 PMC5711979 28889247

[CR5] Bates K, Sweeting J, Yeates L, McDonald K, Semsarian C, Ingles J (2019) Psychological adaptation to molecular autopsy findings following sudden cardiac death in the young. Genet Med 21:1452–1456. 10.1038/s41436-018-0338-4 30327538

[CR6] Burns C, Yeates L, Spinks C et al (2017) Attitudes, knowledge and consequences of uncertain genetic findings in hypertrophic cardiomyopathy. Eur J Hum Genet 25:809–815. 10.1038/ejhg.2017.66 PMC5520078 28594412

[CR7] Couper K, Putt O, Field R et al (2020) Incidence of sudden cardiac death in the young: A systematic review. BMJ Open 10:e040815. 10.1136/bmjopen-2020-040815 PMC7542928 33033034

[CR9] de Noronha SV, Behr ER, Papadakis M, Ohta-Ogo K, Banya W, Wells J et al (2014) The importance of specialist cardiac histopatho-logical examination in the investigation of young sudden cardiacdeaths. Europace 16:899–907. 10.1093/europace/eut329 24148315

[CR8] Deignan JL, De Castro M, Horner VL et al (2023) Points to consider in the practice of postmortem genetic testing: A statement of the American College of Medical Genetics and Genomics (ACMG). Genet Med 25(5):100017. 10.1016/j.gim.2023.100017 36799919

[CR10] Eckart RE, Shry EA, Burke AP et al (2011) Sudden Death in Young Adults: An Autopsy-Based Series of a Population Undergoing Active Surveillance. J Am Coll Cardiol 58(12):1254–1261. 10.1016/j.jacc.2011.01.049 21903060

[CR11] Fellmann F, van El CG, Charron P et al (2019) European recommendations integrating genetic testing into multidisciplinary management of sudden cardiac death. Eur J Hum Genet 27:1763–1773. 10.1038/s41431-019-0445-y PMC6870982 31235869

[CR12] Guo L, Torii S, Fernandez R et al (2021) Genetic variants associated with unexplained sudden cardiac death in adult White and African American individuals. JAMA Cardiol 6(9):1013–1022. 10.1001/jamacardio.2021.1573 PMC8173469 34076677

[CR13] Hofer F, Fellmann F, Schlapfer J, Michaud K (2014) Sudden cardiac death in the young (5–39 years) in the canton of Vaud, Switzerland. BMC Cardiovasc 14:140. 10.1186/1471-2261-14-140 PMC4198689 25291980

[CR14] Katritsis DG, Gersh BJ, Camm AJ, A Clinical Perspective on Sudden Cardiac Death (2016) Arrhythm Electrophysiol Rev 5(3):177–182. 10.15420/aer.2016:11:2 PMC5248660 28116082

[CR15] Landry CH, Allan KS, Connelly KA, Cunningham K, Morrison LJ, Dorian P (2017) Sudden cardiac arrest during participation in competitive sports. N Engl J Med 377:1943–1953. 10.1056/NEJMoa1615710 PMC5726886 29141175

[CR16] Li Tan SM, Lim SL, Ong ME, Leong KM (2025) Genetics of Sudden Cardiac Arrest: Overview of Genetic Risk Factors and Aetiologies. Arrhythm Electrophysiol Rev 14:e18. 10.15420/aer.2025.11 PMC12409730 40918690

[CR17] Lynge TH, Albert CM, Basso C et al (2024) Autopsy of all young sudden death cases is important to increase survival in family members left behind. Europace 26(6):euae128. 10.1093/europace/euae128 PMC11164113 38715537

[CR18] Mellor G, Raju H, de Noronha SV, Papadakis M, Sharma S, BehrER et al (2014) Clinical characteristics and circumstances of death in thesudden arrhythmic death syndrome. Circ Arrhythm Electrophysiol 7:1078–1083. 10.1161/CIRCEP.114.001854 25262685

[CR19] Monda E, Montuoro S, Crotti L et al (2026) Yield of postmortem genetic testing in sudden arrhythmic death syndrome: a systematic review and meta-analysis. Circ Genom Precis Med 19(3). 10.1161/CIRCGEN.125.005523 41979508

[CR21] Ponmani P, Sangita M, Yadav J (2026) A meta-analytic and meta-regression study of postmortem genetic analysis in sudden death: A step towards precision forensic genomics. Forensic Sci Int 383:112893. 10.1016/j.forsciint.2026.112893 41747495

[CR22] Richards S, Aziz N, Bale S, Bick D, Das S, Gastier-Foster J et al (2015) Standards and guidelines for the interpretation of sequence variants: a joint consensus recommendation of the American College of Medical Genetics and Genomics and the Association for Molecular Pathology. Genet Med 17(5):405–424. 10.1038/gim.2015.30 PMC4544753 25741868

[CR23] Semsarian C, Medi C (2025) Screening families after sudden cardiac death in the young: do we ever stop? Europace 27(7):euaf141. 10.1093/europace/euaf141 PMC12264590 40667716

[CR24] Stiles MK, Wilde AAM, Abrams DJ et al (2020) APHRS/HRS expert consensus statement on the investigation of decedents with sudden unexplained death and patients with sudden cardiac arrest, and of their families. J Arrhythm 37(3):481–534. 10.1002/joa3.12449 PMC8207384 34141003

[CR25] van der Werf C, van Langen IM, Wilde AA (2010) Sudden death in the young: what do we know about it and how to prevent? Circ Arrhythm Electrophysiol 3:96–104. 10.1161/CIRCEP.109.877142 20160177

[CR26] Wemeijer TM, Pries AM, Puiman PJ et al (2025) De NODOV-Studie: Een Stap op Weg naar Gestructureerd Postmortaal Onderzoek bij Onverwacht en Onverklaard Overlijden van Jongvolwassenen. TSG Tijdschr Gezondheidswet 103:33–36. 10.1007/s12508-025-00460-6 PMC12185550 40567350

