# Supplementary material for: Structured postmortem genetic counselling and testing in sudden unexplained death among young adults in the Netherlands: results and implications regarding hereditary risk
Source: J Community Genet. 2026 Oct 17;17(6):120. doi: 10.1007/s12687-026-00946-z (PMC13676581; doi:10.1007/s12687-026-00946-z)
Supplement: Supplementary file 1 [file 12687_2026_946_MOESM1_ESM.docx]

**Supplementary Materials**

**Supplement 1.** Summary of conventional autopsy, (postmortem) genetic testing and family recommendations. In general, most cases utilized phenotype-guided strategies. Other test strategies are indicated: † targeted gene mutation known within the extended family; ‡ Affected relatives detected pathological variant later confirmed in the deceased; ∆ Deceased not tested due to known pathological variant. Variant of Unknown significance (VUS) explained: ■ Risk factor associated with atherosclerosis; ◊ VUS with strong molecular suspicion.

| **Case no.** | **Gender** | **Age group** | **Conventional**  **Autopsy** | **(Postmortem) genetic testing** | **Family recommendations** |
| --- | --- | --- | --- | --- | --- |
| 1 | Female | 26–29 | Negative | † Pathogenic CACNA1C gene variant  c.2575C>T, p.(Pro859Ser) | Genetic testing and regular cardiac examination |
| 2 | Male | 26–29 | Negative | Negative | 1x Cardiac examination |
| 3 | Male | 38–41 | Coronary artery disease  Myocardial infarction | Negative | CVRM |
| 4 | Female | 34–37 | Negative | Negative | CVRM |
| 5 | Male | 30–33 | Cardiomyopathy | DNA insufficient | Regular cardiac examination |
| 6 | Female | 22–25 | Negative | Negative | 1x Cardiac examination |
| 7 | Male | 26–29 | Negative | Negative | Regular cardiac examination and CVRM |
| 8 | Male | 30–33 | Coronary artery disease  Myocardial infarction  Infectious | Negative | 1x Vascular internal specialist |
| 9 | Male | 34–37 | Cardiomyopathy  Coronary artery disease | Pathogenic BAG3 gene variant c.346del, p.(Gln116Serfs*95) | Genetic testing and regular cardiac examination |
| 10 | Male | 18–21 | Negative | Permission denied | N/A |
| 11 | Male | 26–29 | Negative | Negative | 1x Cardiac examination |
| 12 | Male | 34–37 | Cardiomyopathy | Negative | Regular cardiac examination |
| 13 | Male | 30–33 | Cardiomyopathy | Negative | Regular cardiac examination |
| 14 | Male | 42–45 | Coronary artery disease | Negative | 1x cardiac examination and CVRM |
| 15 | Male | 34–37 | Multiorgan failure | Negative | Provocation test and 1x cardiac examination |
| 16 | Male | 38–41 | Coronary artery disease  Polycystic liver | Negative | 1x Cardiac examination |
| 17 | Male | 38–41 | Cardiomyopathy | Pathogenic MYBPC3 gene variant, c.2864_2865del, p.(Pro955Argfs*95) | Genetic testing and regular cardiac examination |
| 18 | Male | 42–45 | Coronary artery disease  Myocardial infarction | ■ LPL gene variant (VUS), c.106G>A p.(Asp36Asn) | Lipid testing and CVRM |
| **Case no.** | **Gender** | **Age group** | **Conventional**  **Autopsy** | **(Postmortem) genetic testing** | **Family recommendations** |
| 19 | Male | 42–45 | Coronary artery disease  Myocardial infarction  Infectious | ∆ Pathogenic LDLR gene variant, c.313+1G>C | Genetic testing and regular cardiac examination |
| 20 | Male | 38–41 | Type A dissection (CT-scan) | ◊ COL3A1-gene VUS | 1x Cardiac examination and CVRM  Revisit test possibilities in 4 years |
| 21 | Male | 38–41 | Coronary artery disease  Myocardial infarction  Infectious | Negative | CVRM |
| 22 | Female | 18–21 | Cardiomyopathy | Negative | Regular cardiac examination |
| 23 | Male | 42–45 | Myocardial infarction without coronary artery disease | DNA insufficient | 1x Cardiac examination |
| 24 | Male | 42–45 | Coronary artery disease  Myocardial infarction | Negative | 1x Lp(a) testing and CVRM |
| 25 | Female | 38–41 | Coronary artery disease  Myocardial infarction | Negative | CVRM |
| 26 | Male | 38–41 | Cardiomyopathy | Negative | Regular cardiac examination |
| 27 | Male | 38–41 | Coronary artery disease  Myocardial infarction | Negative | CVRM |
| 28 | Female | 42–45 | Coronary artery disease  Myocardial infarction | Negative | 1x cardiac examination and CVRM |
| 29 | Male | 42–45 | Coronary artery disease | Negative | 1x Cardiac examination and CVRM |
| 30 | Male | 26–29 | Cardiomyopathy  Valvular disease | Negative | Regular cardiac examination |
| 31 | Male | 30–33 | Pulmonary embolism | Not tested | Medication advice for females |
| 32 | Male | 26–29 | Negative | Negative | 1x Cardiac examination |
| 33 | Male | 30–33 | Negative | Pathogenic KCNJ2-gene variant, c.935G>A; p.(Arg312His) | Genetic testing and regular cardiac examination |
| 34 | Male | 30–33 | Negative  SUDEP | Negative | Regular cardiac examination |
| 35 | Male | 26–29 | Cardiomyopathy | Negative | Regular cardiac examination |
| 36 | Male | 26–29 | Brain aneurysm (CT-scan) | Negative | 1x Neurological examination  Revisit test possibilities in 3 years |
| 37 | Female | 38–41 | Coronary artery disease  Myocardial infarction | Negative | CVRM |
| 38 | Male | 18–21 | Negative | Negative | 1x Cardiac examination |
| 39 | Male | 42–45 | Coronary artery disease | Negative | CVRM |
| 40 | Male | 34–37 | Negative | Negative | Regular cardiac examination |
| **Case no.** | **Gender** | **Age group** | **Conventional**  **Autopsy** | **(Postmortem) genetic testing** | **Family recommendations** |
| 41 | Male | 42–45 | Cardiomyopathy | Negative | Regular cardiac examination |
| 42 | Male | 38–41 | Coronary artery disease | Negative | 1x cardiac examination and CVRM |
| 43 | Female | 38–41 | Brain aneurysm  Polycystic kidneys and liver | Pathogenic PKD1-gene variant, c.856_862del p. (Gly287*) in exon 5 | Genetic testing  1x Ultrasound examination |
| 44 | Male | 34–37 | Coronary artery disease  Myocardial infarction | Negative | 1x Vascular internal specialist  Revisit test possibilities in 5 years |
| 45 | Female | 30–33 | Negative | Negative | No follow-up |
| 46 | Male | 42–45 | Coronary artery disease | ■ Lipoprotein(a)-gene variant (VUS), c.3947+467T>C | 1x Lp(a) testing and CVRM |
| 47 | Female | 34–37 | Coronary artery disease  Myocardial infarction  Infectious | Negative | CVRM |
| 48 | Male | 38–41 | Coronary artery disease | Negative | Regular cardiac examination |
| 49 | Male | 34–37 | Coronary artery disease  Myocardial infarction | Negative | CVRM |
| 50 | Male | 42–45 | Cardiomyopathy  Coronary artery disease  Myocardial infarction  Infectious | Negative | Regular cardiac examination and CVRM |
